# Dental Pulp Stem Cell-Derived Conditioned Medium Alleviates Subarachnoid Hemorrhage-Induced Microcirculation Impairment by Promoting M2 Microglia Polarization and Reducing Astrocyte Swelling

**DOI:** 10.1007/s12975-022-01083-8

**Published:** 2022-10-01

**Authors:** Ling-Yu Yang, Yong-Ren Chen, Jing-Er Lee, Kuo-Wei Chen, Hui-Tzung Luh, Yi-Tzu Chen, Kuo-Chuan Wang, Sung-Tsang Hsieh

**Affiliations:** 1 Division of Neurosurgery, Department of Surgery, National Taiwan University Hospital, Taipei, Taiwan; 2 Non-Invasive Cancer Therapy Research Institute, Taipei, Taiwan; 3 Department of Neurology, Taipei Medical University-Wan Fang Hospital, Taipei, Taiwan; 4 Division of Neurosurgery, Department of Surgery, National Taiwan University Hospital Hsin-Chu Branch, Hsin-Chu, Taiwan; 5 Graduate Institute of Clinical Medicine, National Taiwan University College of Medicine, Taipei, Taiwan; 6 Department of Anatomy and Cell Biology, College of Medicine, National Taiwan University, Taipei, Taiwan; 7 Department of Neurology, National Taiwan University Hospital, Taipei, Taiwan

**Keywords:** Aneurysmal subarachnoid hemorrhage, Dental pulp stem cells, Conditioned medium, Microcirculation impairment, Brain edema, Neuroinflammation

## Abstract

Aneurysmal subarachnoid hemorrhage (SAH) can cause severe neurological deficits and high mortality. Early brain edema following SAH contributes to the initiation of microcirculation impairment and may further lead to delayed ischemic neurologic deficit (DIND). This study aimed to investigate whether dental pulp stem cell conditioned medium (DPSC-CM) ameliorates SAH-induced microcirculation impairment and the underlying mechanisms. SAH was induced via intrathecal injection of fresh autologous blood in Wistar male adult rat. DPSC-CM or DPSC-CM + insulin growth factor-1 (IGF-1) antibody was randomly administered by intrathecal route 5 min after SAH induction. To evaluate the underlying mechanisms of DPSC-CM in the treatment of SAH, primary rat astrocyte and microglia co-cultures were challenged with hemolysate or SAH-patient CSF in the presence or absence of DPSC-CM. The results showed that in vivo, DPSC-CM treatment decreased the brain water content, improved microcirculation impairment and enhanced functional recovery at 24 h post-SAH. DPSC-CM treatment also alleviated the expressions of water channel protein aquaporin-4 (AQP4) and pro-inflammatory cytokines, and enhanced the expressions of anti-inflammatory factors in the cortical region. However, all the beneficial effects of DPSC-CM were abrogated after treatment with IGF-1 neutralizing antibody. The in vitro results further showed that DPSC-CM treatment reduced hemolysate/SAH-patient CSF-induced astrocyte swelling and promoted M2 microglia polarization, partially through IGF-1/AKT signaling. The data suggested that DPSC-CM significantly reduced brain edema and rescued microcirculation impairment with concomitant anti-inflammatory benefits after SAH, and may potentially be developed into a novel therapeutic strategy for SAH.

**Supplementary Information:**

The online version contains supplementary material available at 10.1007/s12975-022-01083-8.

## Introduction

Spontaneous subarachnoid hemorrhage (SAH) accounts for approximately 5% of all strokes, and ruptured aneurysms cause approximately 85% of SAH cases [1]. Although aneurysmal SAH is less frequent than ischemic stroke, it is a devastating disease that affects young people and is associated with long-term neurological deficits and high mortality [2]. Accumulating evidence shows that early brain injury (EBI), such as brain edema, brain tissue hypoxia, and blood–brain barrier (BBB) breakdown, often occurs within 72 h after SAH and contributes to cerebral vasospasm or delayed ischemic neurologic deficit (DIND) in the subacute phase [3].

Global cerebral edema is observed in a number of patients (8–67%) and often occurs within the first 24 h after SAH, which has been recognized as a major predictor of a poor outcome [4]. Cytotoxic edema refers to cellular swelling, in most cases occurring within minutes to hours of an acute inflammation insult, and particularly seen in astrocytes. Increased swelling of astrocytic end-feet, which then compress the capillary lumen, was observed in a rodent model of SAH [5]. Multiple lines of evidence suggest that upregulation of the AQP4 expression, which is predominantly found in capillaries surrounding astrocyte end-feet, is associated with cytotoxic edema formation [6]. Currently, available treatments for cerebral edema are limited to hypothermia, osmotherapy and surgical decompression, which are usually administered based on symptoms and often lead to adverse side effects [7]. Therefore, it is necessary to develop a novel effective therapy to overcome cytotoxic edema in EBI, which may help to improve the impaired microcirculation and reduce subsequent delayed edema formation.

A variety of mechanisms are thought to be involved in the pathogenesis of cytotoxic edema following SAH. When blood derived from the rupture of an aneurysm leaks into the subarachnoid space, erythrocyte hemolysis, and consequent release of oxyhemoglobin caused free radicals and thrombin-induced inflammatory cytokine generation [8]. Meanwhile, the inflammatory response after SAH initiates a cascade of immune cell activation in the brain, such as microglia, the predominant immune cells in the brain, and infiltration of leukocytes, which may contribute to cytotoxic edema formation [9]. Multiple lines of evidence suggest that the microglia-mediated immune response and upregulated pro-inflammatory cytokines, such as IL-6, IL-1β, and TNF-α, appear to represent an independent risk factor for brain edema after SAH [10–12]. One study demonstrated that modulation of microglial activation from a pro-inflammatory (M1) to an anti-inflammatory (M2) phenotype via mTOR inhibition could reduce brain edema formation in a rat SAH model [10]. In addition, previous studies indicated that proinflammatory M1 microglia secreted cytokines, such as TNFα, exert influence on astrocyte activation and AQP4 expression [13, 14]. Thus, the immunomodulation of microglia towards an anti-inflammatory (M2) phenotype might be an important therapeutic strategy by which to prevent early brain edema formation and subsequent brain injury in this highly morbid condition.

Growing evidence have demonstrated that the paracrine factors derived from mesenchymal stem cells (MSCs) may play important roles in intercellular communication and contribute to neuroprotective and anti-inflammation properties [15, 16]. Activated MSCs respond to TLR ligands and exert immunomodulatory functions, including inhibition of T-cell/B-cell proliferation and differentiation through paracrine mechanisms [17]. This involves the production of anti‐inflammatory factors, such as IL-10, TGF-β1, insulin-like growth factor 1 (IGF-1), microvesicles, and exosomes [18]. Systemic administration of MSC-derived extracellular vesicles [19] and conditioned medium [20] has been shown to improve functional recovery in rodent models of cerebral ischemia. Clinical application of such cell-free approaches is attracting growing interest, as they avoid certain risks related to stem-cell therapy, including tumorigenicity, virus contamination, and others [21]. However, there is a still lack of evidence regarding the benefits of cell-free therapy for SAH.

Recently, we have successfully built a dental pulp stem cell (DPSC) culture system and found that serum-free DPSC-conditioned medium (DPSC-CM) has therapeutic effects in terms of alleviation of neuroinflammation and neurological deficits after experimental SAH induction in rats [22]. Our study unveiled the most abundant protein in DPCS-CM was found to be IGF-1 (42.64%), followed by TIMP-2 (39.03%), TIMP-1 (11.66%), TGF-β (6.55%), and others (0.12%) [22]. IGF-1 has been implicated in several anti-oxidative, anti-inflammatory, and anti-apoptotic protective roles in brain injury and edema [23, 24]. In the present study, we aimed to evaluate the therapeutic effects of DPSC-CM on early brain edema formation and microcirculation impairment in an in vivo experimental rat model of SAH. We also developed an in vitro model of primary astrocyte-microglia co-cultures to evaluate the role of DPSC-CM and IGF-1 in hemolysate-induced astrocyte swelling and M2 microglia polarization.

## Materials and Methods

### Ethics Statement

This research program included the performance of animal experiments and obtaining CSF from human subjects (hCSF), as detailed below, and was reviewed and approved by the Institutional Animal Care and Use Committee of National Taiwan University and the Institutional Review Board of National Taiwan University Hospital, Taipei, Taiwan. Animals were housed in groups in a temperature- (21 ~ 25 °C) and humidity-controlled (45 ~ 50%) room with a 12-h light/dark cycle with ad libitum access to food and water. All animal protocols were performed in accordance with the guidelines of the Animal Welfare Protection Act of the Department of Agriculture, Executive Yuan, Taiwan.

### Preparation of DPSC-CM

DPSC-CM collection was performed according to our previous report [22]. DPSCs were isolated from 3-week-old male Wistar rats and cultured in alpha-MEM supplemented with 10% FBS (Gibco). The DPSCs (P3) exhibited a fibroblastic morphology and were previously characterized as MSCs via flow cytometry analysis of surface markers (CD44, CD90, CD73, and CD105), but not endothelial/hematopoietic markers such as CD34 and CD45 [22]. Conditioned medium (CM) was generated from the DPSCs (P3–P5), which were cultured in a 150-cm^2^ flask and fed with 10 mL DMEM/F12 and 10 mL PBS, then incubated for 48 h. At 80–90% confluence, the medium was collected and centrifuged for 3 min at 2500 rpm. For the in vitro and in vivo experiments, the CM was further concentrated using a tangential flow filtration (TFF) membrane filter system unit with a 5–30-kDa cutoff (Millipore, Burlington, MA, USA) following the manufacturer’s instructions. The total proteins in the 500 ml DPSC-CM was 17.71 mg (35.42 µg/ml), while the amounts of 5–30 kDa was 3.61 mg (20.4%). As for different rat DPSC-CM preparations, the variation of protein amounts within ± 10% in each group were be used for the quality control.

### In vivo Studies

#### Animal Model of SAH

An animal model of SAH was induced in adult male Wistar rats (weighing 250 ~ 300 g). The rats were anesthetized with 2.5% isoflurane with 70% nitrous oxide and 27.5% oxygen. The animals were randomized into four groups: (i) sham, (ii) SAH + Veh, (iii) SAH + DPSC-CM, and (iv) SAH + DPSC-CM + anti-IGF-1 protein (PEPROTECH, 500-P11). The SAH induction procedure was performed as described previously [22, 25]. Briefly, an incision was made in the suboccipital region and the atlanto-occipital membrane overlying the cisterna magna was exposed. After treatment, SAH was induced via intrathecal injection of fresh autologous blood (0.3 ml) from the femoral artery into the cisterna magna, and the animal’s head was placed at a 20° head-down position over a period of 2 to 3 min. After suturing the skin, the body temperature was monitored with a rectal probe and maintained at 37.0 ± 0.5 °C using a heated pad until recovery from anesthesia. A right femoral arterial catheter was employed for continuous monitoring of blood pressure and maintained at 100–120 mmHg using an RFT Biomonitor, VEB (Messgeraetewerk, Germany).

#### Treatment Algorithm

The sham and SAH group animals received an intrathecal injection of 40 μl vehicle (normal saline). To determine the most efficacious dose of DPSC-CM, a low dose (20 μl/rat) or high dose (40 μl/rat) of DPSC-CM was received an intrathecal injection at 10 min before SAH induction. As Supplementary Fig. 1 shows, only high dose (40 μl/rat) of DPSC-CM displayed significant improvement on microcirculation than that in vehicle-treated rats at 24 h after SAH induction. We therefore selected the high dose (40 μl/rat) of DPSC-CM or DPSC-CM + anti-IGF-1 protein (40 μl, anti-IGF-1 antibody 100 μg mixed with 3 ml DPSC-CM) for the following treatment groups. Notably, no temperature changes were observed after vehicle, DPSC-CM, or DPSC-CM + anti-IGF-1 protein treatment over a period of 3 h. All handling and processing of tissues was carried out by researchers blind to the treatment.

#### Microcirculation Assessment

Observation and quantification of brain surface microcirculation at 24 h and 48 h after SAH was performed as described previously [22, 26]. Briefly, the dura mater was removed after a left frontal craniotomy. A video capillaroscopy (CAM1 Capillary Anemometer, KK Technology, England) with a high-resolution (752 × 582 pixels) monochrome charge-coupled device (CCD) video camera was used to measure the capillary microcirculation on the rat brain surface. Each field of the craniotomy site with 1–2 main arterioles was observed and recorded. To quantify the microcirculation, the main arteriole was divided into primary arterioles (pa), followed by secondary arterioles (sa), and finally terminal arterioles (ta) according to the branch order. We first identified the main arterioles, then took photographs along the pa, sa, and ta until communication with the territory of another main arteriole. Each photographic field covered approximately 3 × 3 mm^2^, and more than 10 photographs were taken. After integrating all photographs to show the entire field of the main arteriole, the diameters of the pa, sa, and ta were measured individually in each animal group.

#### Measurement of Brain Regional Blood Flow and Partial Pressure of Oxygen in Brain Tissue

To measure the tissue perfusion and the partial pressure of oxygen (PbtO_2_) in the rat cortex, animals subjected to SAH induction and treated with DPSC-CM or DPSC-CM + IGF-1 neutralizing antibody were evaluated using OxyLite 2000E and OxyFLO 2000E detectors (Oxford Optronic Ltd, England) at 24 h and 48 h after SAH [22]. Rats were anesthetized and fixed in a stereotactic apparatus, and the PbtO_2_, blood flow, and temperature in the cortex were recorded simultaneously at the same tissue micro-region.

#### Transmission Electron Microscopy (TEM)

TEM was performed using the electron microscope equipment of the Pathology Department of National Taiwan University Hospital (Taipei, Taiwan). Brain slices of 1 mm containing the gray matter were collected and pre-fixed in PBS with 2.5% glutaraldehyde, then post-fixed with 2% osmium tetroxide for 1 h. Then, the brain samples were dehydrated in graded ethanol and embedded in epoxy resin. Ultra-thin sections were cut and placed on a copper grid stained with uranyl acetate and lead citrate. Specimens at a magnification of 1500 × /8000 × were obtained using a high-resolution transmission electron microscope (JEOL JEM-1400, Japan) at 80 kV.

#### Brain Water Content

At 24 h following SAH induction, rats were sacrificed and their brains quickly removed. The brain tissue was divided into the following three parts: cortex, brain stem, and cerebellum. Each part of the brain was weighed immediately after cutting (wet weight) and then dried in an oven at 80 °C for 3 days to achieve complete desiccation and re-weighed to obtain a dry weight. The brain water content was calculated as (wet weight − dry weight)/wet weight × 100% [27].

#### Rotarod Test

The rotarod test is often used to measure general motor activity [28]. We performed the rotarod test as previously described [22]. In the training phase, each rat was placed on the instrument (Panlab Rota Rod, USA) at a speed of 4 rpm for three consecutive days, three sessions per day for 5 min. The latency to fall of each animal was recorded at a speed of 4–40 rpm, in 600 s, during a 5-min testing period 7 days after SAH.

#### Enzyme-Linked Immunosorbent Assay (ELISA)

Plasma from the various groups of animals was collected at 24 h after SAH and stored at − 80 °C. The samples were assayed in duplicate using IL-10 assay kits (Abnova Systems, USA), according to the manufacturer’s guidelines. The concentration of protein is expressed as picograms of antigen per milligram of protein.

### In vitro Studies

#### Primary Rat Mixed Astrocytes and Microglia Cell Cultures

Mixed astrocytes and microglia co-cultures were prepared from the cerebral cortex of 1–3-day-old neonatal Wistar rats based on modifications of a method described previously [29]. The brain cortical tissues were collected in ice-cold Ca/Mg-free HBSS (Biological Industries, Israel). The meninges were removed and then cortical cells were dissociated by trituration using a pipette. A cell pellet was obtained by centrifugation (at 1500 rpm for 5 min) and re-suspended in Dulbecco’s modified Eagle’s medium (Corning, USA) with 10% FBS, 1% penicillin, and 0.25% gentamycin (Biological Industries, Israel). The cell suspension was seeded at a density of 5 × 10^5^ cells/ml and added to 10-cm culture dishes (10 ml/dish), then incubated at 37 °C in humidified 5% CO_2_/95% air. The culture medium was replenished 4 days after plating, and changed every 3 days thereafter. All experiments were performed 15–20 days after plating. Immunostaining for the astrocyte-specific marker glial fibrillary acidic protein (GFAP) (ab7260, Abcam) and macrophage/microglia-specific protein Iba1 (ab5076, Abcam) was used to determine the percentage of cell composition, followed by cell counting. The mixed glia cultures consisted of approximately 75 ~ 85% astrocytes and 15 ~ 25% microglia.

#### Preparation of Hemolysate

Erythrocyte hemolysis plays an important role in the development of vasoconstriction after SAH and has been used as an in vitro model of SAH [30, 31]. Hemolysate preparation by a rapid freeze–thaw method to lyse red blood cells has been described previously and was followed in this study with minor modifications [30]. Blood samples were collected using a heparinized sterile syringe via cardiac puncture in rats. The blood was poured into 15-ml centrifuge tubes then centrifuged at 2500 × *g* for 15 min at 4 °C. The supernatant was then removed and discarded, and the red blood cells re-suspended in an equal volume of sterile distilled water. The red blood cells were then frozen at − 80 °C for 30 min and subsequently rapidly thawed in a 39 °C water bath. The cells were then centrifuged at 14,000 × *g* for 30 min at 4 °C. This process lysed the cells and produced hemolysate in the supernatant, which was collected and stored at − 80 °C. Hemolysate concentration = (hemolysate mass − distilled water mass)/hemolysate volume. Treatment with 1 mg/ml hemolysate for 24 h in primary glial cell cultures was used as an in vitro SAH model, as described in a previous study [30].

#### Collection of CSF from SAH Patients

CSF collection from SAH patients has been described previously [25]. We employed CSF from SAH patients with an unfavorable outcome in a mixed glia culture as a subacute or late-stage SAH in vitro model. Intrathecal CSF was collected via lumbar puncture on the seventh day after SAH based on our previously described protocol [32]. The CSF samples were immediately centrifuged at 900 × *g* at 4 °C for 20 min before being divided into suitable aliquots and snap-frozen at – 80 °C within 30 min. Written informed consent was obtained from all patients or their legal representatives for this study.

#### Treatment of Cells

The mixed glia cultures were randomly assigned into eight groups: (i) PBS-treated control group; (ii) hemolysate (1 mg/ml) treatment group; (iii) hemolysate (1 mg/ml) with DPSC-CM (20%) treatment group; (iv) hemolysate (1 mg/ml) with DPSC-CM (20%) + anti-IGF-1 protein (0.2 μg/ml) treatment group; (v) hemolysate (1 mg/ml) with DPSC-CM (20%) + LY294002 (10 μM) treatment group; (vi) SAH-patient CSF (10%) treatment group; (vii) CSF (10%) with DPSC-CM (20%) treatment group; (viii) CSF (10%) with DPSC-CM (20%) + anti-IGF-1 protein (0.2 μg/ml) treatment group. PI3K/Akt inhibitor, LY294002, were obtained from Sigma-Aldrich, USA, and dissolved in DMSO. All cells were incubated for 24 h at 37 °C, 5% CO_2_.

#### Measurement of the Cell Perimeter

In order to further evaluate the volume change of astrocytes, we used the perimeter method, which has been proven to be accurate for measuring volume changes [33]. The cell perimeter was calculated using ImageJ software after GFAP immunofluorescence staining [34]. We randomly selected 10 cells in each field of view at high magnification (200 ×). Five fields were measured in every group, and the average value of the five fields was taken as the cell perimeter of each group.

#### Immunohistochemistry (IHC), Immunocytochemistry (ICC), and Immunofluorescence (IF)

Serial coronal brain Sects. (6 μm) were used for double-fluorescent immunohistochemistry staining. Brain sections were permeabilized and then incubated with PBS containing 3% bovine serum albumin for 1 h at room temperature. Sections were subsequently incubated with a primary antibody overnight at 4 °C. Mouse polyclonal anti-AQP4 (ab9512, Abcam) or mouse polyclonal anti-4-HNE (MAB3249, R&D Systems) were determined by co‑labeling with an antibody against rabbit polyclonal anti-GFAP (ab7260, Abcam) at 24 h following SAH induction. After incubation at 4 °C overnight, the sections were then washed and incubated with Alexa Fluor 488 goat anti-rabbit IgG (111–545-144, Jackson) or Alexa Fluor 594 goat anti-mouse IgG (115–585-003, Jackson) at room temperature for 30 min, and counterstained with DAPI (blue). Sections were then mounted using Mounting Medium H-1000 (Vector Laboratories).

Mixed astrocytes and microglia co-cultures were fixed with 4% paraformaldehyde for 20 min and permeabilized. After blocking with 3% bovine serum albumin for 1 h at room temperature, the cells were incubated overnight at 4 °C with the following primary antibodies: (i) mouse monoclonal CD11b (OX-42, ab1211; Abcam); (ii) rabbit polyclonal anti-arginase-1 (Arg-1) (93668, Cell Signaling Technology); (iii) rabbit polyclonal GFAP (ab7260; Abcam); (iv) anti-4-HNE (MAB3249, R&D Systems), followed by Alexa Fluor 488 goat anti-rabbit IgG (111–545-144, Jackson) and Alexa Fluor 594 anti-mouse IgG (115–585-003, Jackson) at room temperature for 30 min and counterstained with DAPI (blue) or the Novolink Polymer Detection System (RE7140-K; Novocastra). All images were acquired using a Nikon Eclipse Ti2 fluorescence microscope attached to a digital camera and Nikon NIS Elements imaging software. Quantification of fluorescence intensity for AQP4/GFAP in the cortex and ascertaining the numbers of OX42/Arg-1- and 4-HNE-positive cells in co-cultures were performed using ImageJ software.

#### RNA Extraction, Reverse Transcription, and Real-Time Quantitative PCR (qPCR)

Total RNA of tissue (approximately 50 mg) or cells were extracted using TRIzol reagent (Invitrogen, USA) according to the manufacturer’s instructions. Two micrograms of RNA were reverse-transcribed to cDNA using M-MLV reverse transcriptase (Promega Corporation, USA) following the manufacturer’s instructions. All qPCR assays were performed using a 7900 Real-Time PCR System (Applied Biosystems, USA) with a Kapa SYBR Fast qPCR Kit (Kapa Biosystems, USA). All the relevant primer sequences used in the qPCR are shown in supplementary Table S1. The reaction was carried out with 40 cycles of amplification at 98 °C for 2 min, 98 °C for 2 s, and 58 °C for 5 s. The levels of IL-6, IL-1β, TNF-α, Arg-1, TGF-β, IL-4, and IL-10 were normalized to that of the house keeping protein Ppib and then analyzed using the comparative threshold cycle (Ct) method.

#### Western Blot Analysis

The cortex region of the brain or the co-cultured cells were harvested and homogenized at 24 h after SAH induction, and total proteins were lysed in protein extraction buffer (iNtRON Biotechnology, Korea) containing protease and phosphatase inhibitors (Roche, Diagnostics, USA). Lysed cells/tissues were centrifuged at approximately 12,500 rpm for 15 min at 4 °C, and the supernatants were stored at – 80 °C for subsequent Western blot analysis. Proteins were separated by electrophoresis using 12% polyacrylamide gel (Bio-Rad, USA) with a tris–glycine running buffer and then transferred to a nitrocellulose membrane (Bio-Rad Laboratories, USA). Membranes were incubated in blocking buffer with 5% non-fat milk for 1 h at room temperature and then incubated overnight at 4 °C with primary antibodies, including those that selectively bind 4-HNE (MAB3249, R&D Systems), AQP4 (ab9512, Abcam), pAKT (4060, Cell Signaling Technology), AKT (4691, Cell Signaling Technology) and GAPDH (MA515738, Thermo Fisher), followed by goat polycolonal anti-rabbit IgG (Gene-Tex, 1:5000) and goat polycolonal anti-mouse IgG (GeneTex, 1:5000) for 1 h at room temperature. The immunoreactions were visualized using enhanced chemiluminescent (ECL) detection reagents, and the expression levels of proteins were normalized to those of GAPDH or β-actin. Protein expression was quantified using ImageJ software.

### Statistical Analysis

The results are expressed as mean ± standard error of the mean (SEM). Statistical significance and bar graph displays across groups were assessed by one-way analysis of variance (ANOVA) followed by a post hoc test (Tukey’s multiple comparisons test) using SigmaPlot 10 and SigmaStat 3.5. (Jandel Scientific Corp., USA). A probability level of *p* < 0.05 or less was considered statistically significant.

## Results

### Astrocyte Swelling-Mediated Brain Surface Microcirculation Impairment at 24 h and 48 h after SAH

**Fig. 1:**
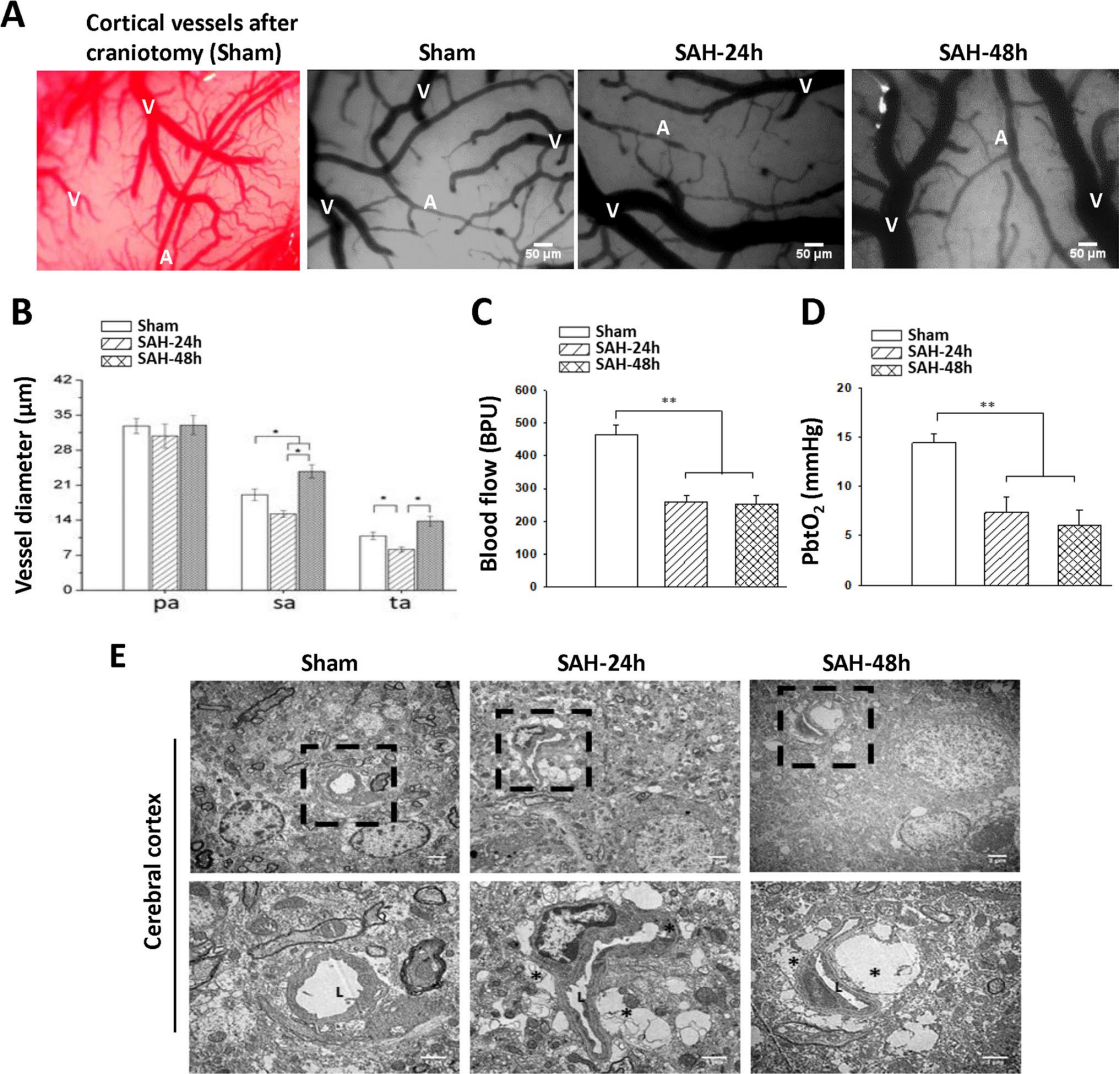
Astrocyte swelling and impaired microcirculation on the cortical surface at 24 h and 48 h after SAH induction. **A** After a craniotomy was performed from the sham, SAH 24 h and SAH 48 h groups, all vasculatures including the main arterioles and venules on the brain surface were clearly seen (A represents arterioles, V represents venules, bar = 50 μm). **B** Observation of the arteriole diameters of primary (pa), secondary (sa), and terminal (ta) arterioles using a CAM1 capillary anemometer. Quantitatively, the diameters of the sa and ta were smaller at 24 h in the SAH group than in the sham group and were increased at 48 h as compared with 24 h after SAH. **C** The regional cerebral blood flow and **D** PbtO_2_ were significantly lower at both 24 h and 48 h in the SAH group than in the sham group. **E** The arrow indicates microvessels in representative electron micrographs (bar = 2 μm). The areas marked by a square are shown in higher magnification of the lower panel (bar = 1 μm). L marks the lumen of a microvessel and asterisks mark the end-feet of astrocytes. The swollen end-feet (*) remarkably compressed the microvessels in the SAH 24 h and SAH 48 h groups. Data are expressed as means ± SEM. *P < 0.05, **P < 0.01, *n* = 4

To investigate cortical surface microcirculation alterations, a craniotomy was performed at 24 h and 48 h after experimental SAH, and all vasculatures including the main arterioles and venules on the brain surface were clearly seen, as shown in Fig. 1A. Arterioles were further divided into primary arterioles (pa), secondary arterioles (sa), and terminal arterioles (ta). Compared with the arterioles in the sham group, markedly diffuse vasoconstriction was observed over the brain surface at 24 h but not at 48 h after SAH induction. Quantification of the diameters of the cortical arterioles showed that the diameters of the sa and ta were significantly reduced as compared with the sham group at 24 h after SAH (Fig. 1B; *P* < 0.05 vs. sham). Notably, as the time increased from 24 to 48 h after SAH, the diameters of the sa and ta were significantly increased (Fig. 1B; *P* < 0.05 vs. SAH 24 h). However, the SAH-induced decrease in cerebral blood flow and PbtO_2_ at 24 h did not improve at 48 h with the increased diameters of arterioles in the animals (Fig. 1C, D). In light of this paradox, we further used electron microscopy to observe the structure of the microcirculation; interestingly, we found that capillaries were dramatically compressed and obstructed by swelling of astrocyte end-feet at 24 h, which persisted at 48 h after SAH induction (Fig. 1E).

### Effects of DPSC-CM on Brain Water Content, Motor Function, and Microcirculation Impairment After SAH

**Fig. 2:**
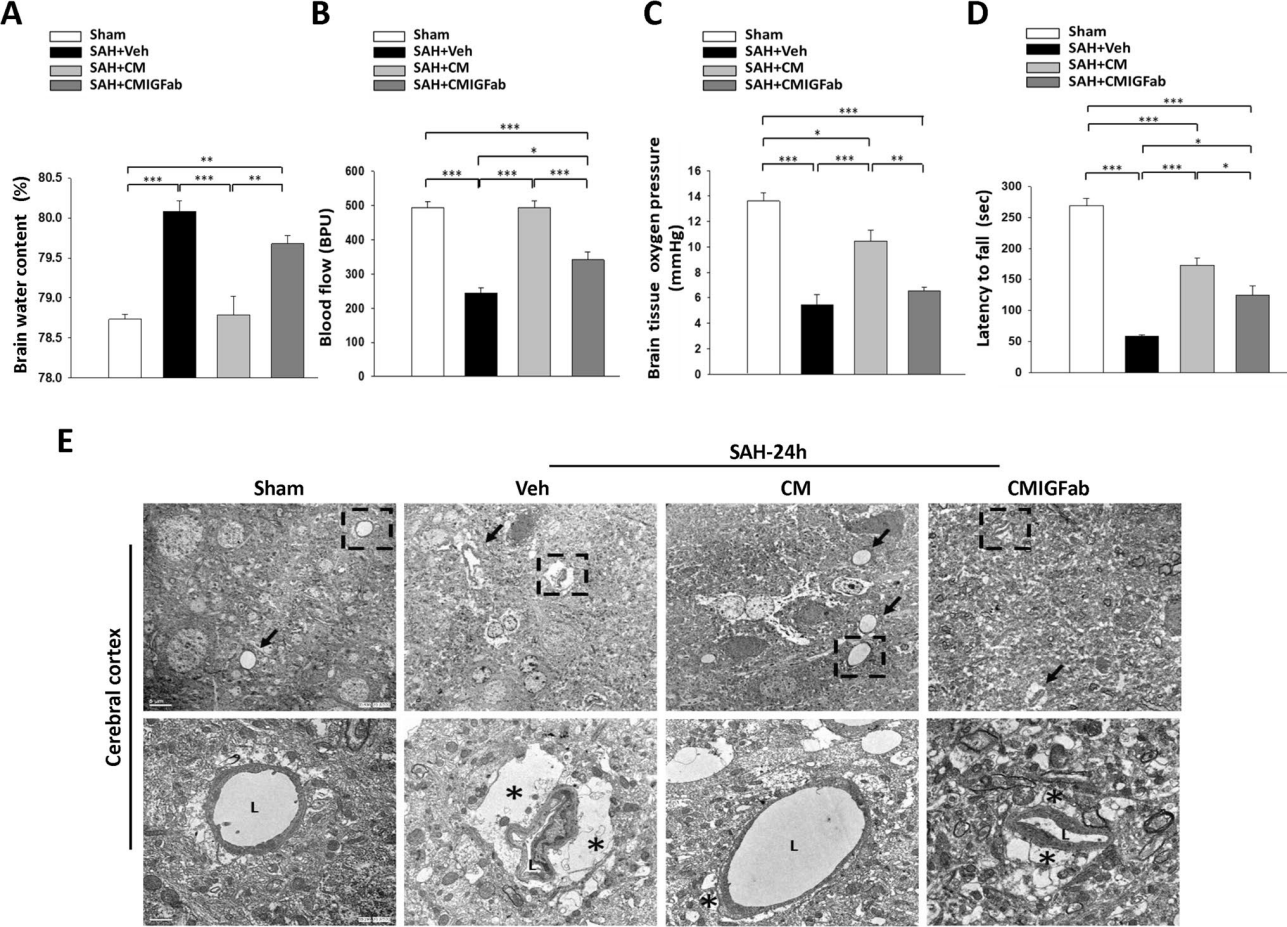
Effects of DPSC-CM on brain edema and microcirculation at 24 h post-SAH. **A** DPSC-CM administration significantly reduced the brain water content in the cortex region at 24 h after SAH. **B** The regional cerebral blood flow and **C** the partial pressure of oxygen (PbtO_2_) at the brain surface were significantly higher in the SAH + CM rats than in the SAH + Veh rats. However, the administration of IGF-1 neutralizing antibodies moderately blunted the DPSC-CM-mediated effects on the two parameters. **D** DPSC-CM significantly improved the latency to fall at 7 days after SAH induction, as measured by the Rotarod test. **E** The arrow points to microvessels in representative electron micrographs (bar = 5 μm) from the four groups. L marks the lumen of a microvessel and asterisks mark the end-feet of astrocytes in the higher-magnification images of the lower panel (bar = 1 μm). The swollen end-feet (*) remarkably compressed the microvessels in the SAH + Veh and SAH + CMIGFab groups, whereas DPSC-CM administration attenuated astrocyte swelling. Data are expressed as means ± SEM. *P < 0.05, **P < 0.01, ***P < 0.001, *n* = 4–5

At 24 h after induction of SAH, we observed a marked increase in cortical water content as compared to the sham group (*P* < 0.001, Fig. 2A). Notably, DPSC-CM treatment significantly attenuated SAH-mediated increases in cortical water content (*P* < 0.001, Fig. 2A). However, the neutralization of IGF-1 antibody moderately abrogated the DPSC-CM-mediated effect (*P* < 0.01, Fig. 2A). Microcirculation assessment including regional blood flow and PbtO_2_ values recorded at a depth of 2 mm from the cortex showed great reductions at 24 h after SAH induction (Fig. 2B, C; *P* < 0.001 vs. sham, respectively). DPSC-CM treatment significantly improved blood flow (*P* < 0.001 vs. SAH + Veh) and oxygen pressure (*P* < 0.05 vs. SAH + Veh), while administration of the IGF-1 neutralizing antibody significantly blunted the treatment efficacy of DPSC-CM in terms of blood flow (*P* < 0.001 vs. SAH + CM) and oxygen pressure (*P* < 0.01vs. SAH + CM) in SAH-injured rats (Fig. 2B, C). The Rotarod test was used to examine the motor coordination at 7 days after SAH induction (Fig. 2D). SAH-injured rats exhibited a lower latency to fall as compared with the sham group (*P* < 0.001), while DPSC-CM treatment significantly improved the latency to fall in comparison with the SAH + Veh group (*P* < 0.001). However, neutralization of IGF-1 antibody abrogated the therapeutic effect of DPSC-CM (*P* < 0.01). Electron microscopy (EM) was used to study the structures of astrocytes and blood vessels at 24 h after SAH (Fig. 2E). In the SAH group, EM revealed that numbers of obstructed microvessels were dramatically compressed and the capillary lumen was narrowed as a result of swelling of astrocyte end-feet, which might cause blood flow restriction in the cortical area. Administration of DPSC-CM attenuated the astrocyte swelling and did not compress the capillary lumen, while the obstructed microvessels and astrocyte end-feet swelling were detected by exposure to IGF-1 neutralizing antibody.

### SAH-Induced Astrocyte Activation and AQP4 Expression in the Cortical Region Were Mitigated by DPSC-CM

**Fig. 3:**
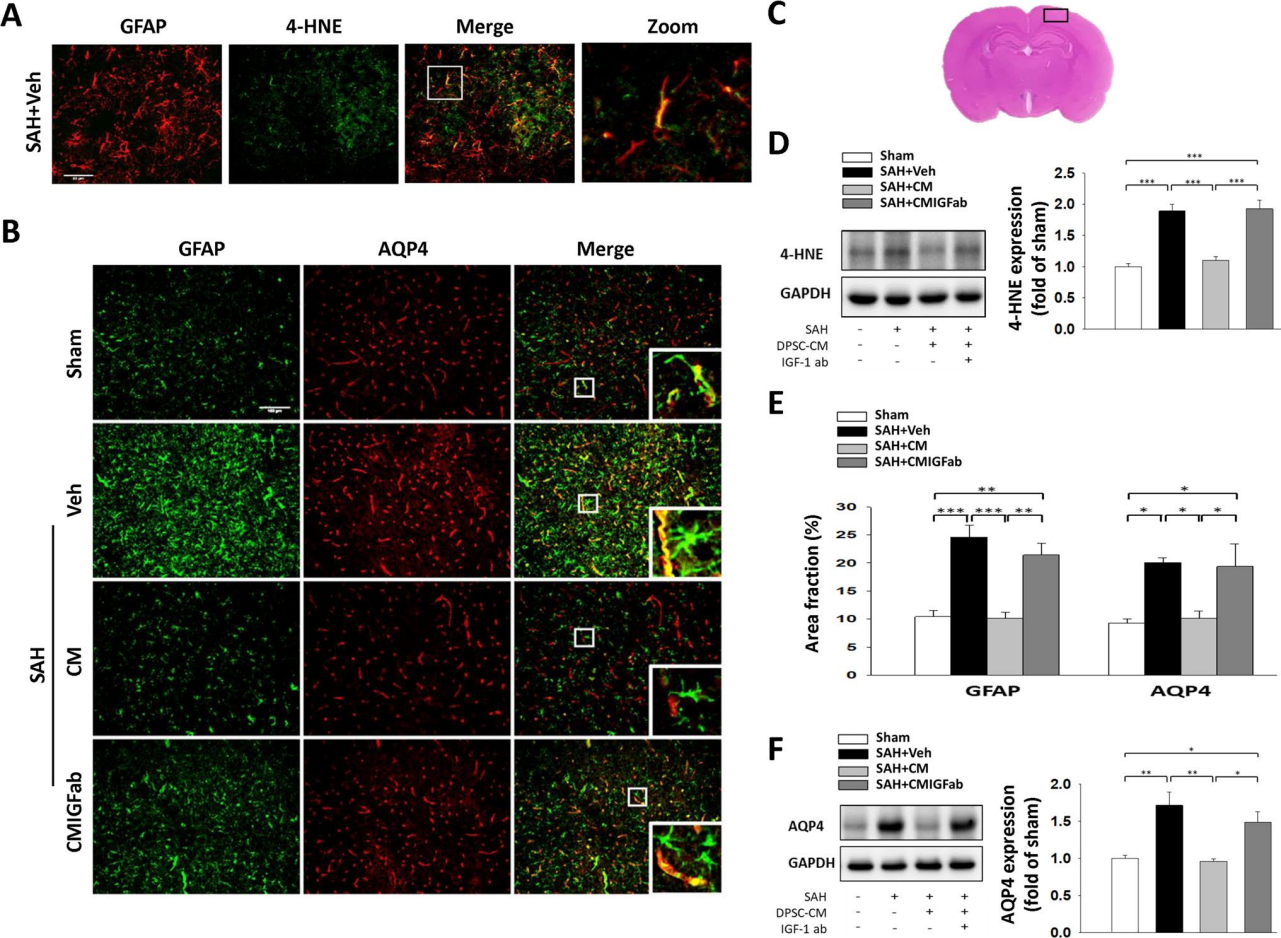
DPSC-CM administration reduced the expression of AQP4 and 4-HNE in astrocyte at 24 h post SAH. **A** Representative immunofluorescence images of 4-HNE (the product of lipid peroxidation; green) and GFAP (a marker for astrocyte; red) labeling in the cerebral cortex region from a SAH animal are shown. Remarkably 4-HNE accumulation was identified in astrocytes after SAH induction (bar = 50 μm). **B** Representative immunofluorescence images of GFAP and AQP4 labeling in the cerebral cortex region. GFAP immunoreactivity is shown in green, and AQP4 is shown in red (bar = 100 μm). **C** Representative HE-stained coronal sections from a sham control showing the cortex region to compare the fluorescent signals between the 4 groups of rats, as indicated by the black square boxes. Western blot analysis showed that DPSC-CM administration reduced the expressions of **D** 4-HNE and **F** AQP4 in the cortical region at 24 h after SAH. **E** GFAP and AQP4 expressions were quantified using the area fraction (percentage of GFAP or AQP4 immunoreactivity in the overall field). Data are expressed as means ± SEM. *P < 0.05, **P < 0.01, ***P < 0.001, *n* = 5

Evidence suggests that oxidative stress plays a role as the one of the factors contributing to post-hemorrhagic brain edema and vasospasm [35]. Double immunofluorescence staining with antibodies towards GFAP and 4-hydroxynonenal (4-HNE, a marker of ROS-dependent lipid peroxidation) demonstrated the presence of 4-HNE in reactive astrocytes within the cortex region at 24 h after SAH (Fig. 3A). The protein levels of 4-HNE following SAH were also measured using Western blot analysis (Fig. 3D). Compared with the sham group, the 4-HNE protein expression was significantly increased at 24 h after SAH induction (*P* < 0.001). Notably, DPSC-CM treatment significantly attenuated the SAH-induced 4-HNE expression increase in the cortex region in comparison with the SAH + Veh group (*P* < 0.001). Given the importance of the role of AQP4 in astrocyte swelling and the development of brain edema, the expression of AQP4 in the cortex was measured via immunofluorescence staining and Western blot at 24 h after SAH (Fig. 3B, E). On histological evaluation of areas within the cortex, GFAP and AQP4 double immunofluorescence staining showed that the percentage of GFAP (*P* < 0.001 vs. sham) and AQP4 (*P* < 0.05 vs. sham) immunoactivites was significantly increased after SAH induction (Fig. 3E). Meanwhile, AQP4 was present on GFAP-positive astrocyte end-feet, indicating the pronounced swelling of reactive astrocytes (Fig. 3B). Our results also showed that DPSC-CM markedly reduced the percentage of GFAP (*P* < 0.001 vs. SAH + Veh) and AQP4 (*P* < 0.05 vs. SAH + Veh) in the cortex region, while the IGF-1 neutralizing antibody abolished the effects of DPSC-CM (Fig. 3E). Furthermore, the Western blot results showed that the level of AQP4 was markedly increased at 24 h after SAH (Fig. 3F, *P* < 0.05 vs. sham). DPSC-CM treatment clearly decreased the expression of AQP4 after SAH (*P* < 0.05 vs. SAH + Veh); however, the effects were reversed by exposure to IGF-1 neutralizing antibody.

### DPSC-CM Treatment Mitigated Neuroinflammation by Promoting Microglia M2 Polarization After SAH Injury in Rats

**Fig. 4:**
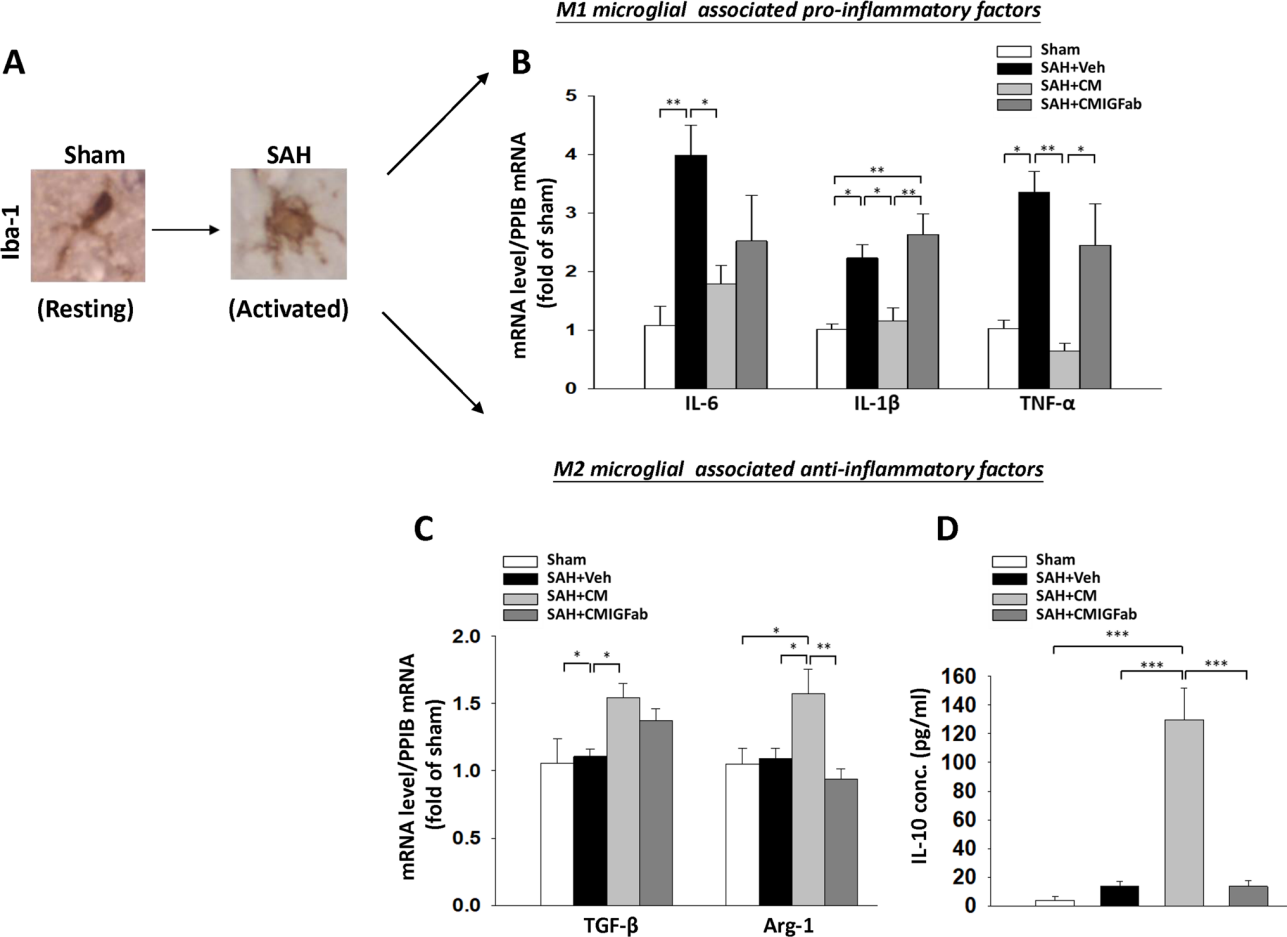
Effects of DPSC-CM on pro- and anti-inflammatory microglial (M1-, M2-like) states at 24 h post-SAH. **A** Representative immunostaining with Iba-1 in the cerebral cortex region and a morphological change from a “resting” form in the sham animal to an “activated” morphology in a SAH animal. **B** Real-time PCR analysis showed that DPSC-CM administration reduced the mRNA expression of M1 microglial-associated pro‐inflammatory factors, including IL-6, IL-1β, and TNF-α. **C** SAH induced the mRNA levels of M2 microglial-associated anti‐inflammatory factors, including TGF-β and Arg-1, which were significantly increased in the DPSC-CM group. **D** ELISA of the protein levels of IL-10 in the four groups showed that DPSC-CM administration significantly increased the IL-10 expression in plasma samples. Data are expressed as means ± SEM. *P < 0.05, **P < 0.01, ***P < 0.001, *n* = 5

Neuroinflammation is another key component of brain edema in EBI and is responsible for poor outcomes after SAH [36]. As illustrated in Fig. 4A, the morphology of Iba-1–immunoreactive microglia in sham animals were ramified and a small cellular body, while the microglia within the SAH group showed a “reactive” morphology with hypertrophied body and shorter or no branching processes. To investigate the roles of DPSC-CM in regulating microglial M1/M2 polarization following SAH, we further examined the expressions of pro-inflammation and anti-inflammation mediating molecules in the cortex at 24 h after SAH. As M1 microglia are known to produce elevated levels of pro-inflammatory cytokines, we evaluated the mRNA expression levels of IL-6, IL-1β, and TNF-α in the cortex (Fig. 4B). Each was significantly elevated within the SAH + Veh group as compared with the sham group. The increases in the expressions of all three pro-inflammatory cytokines after SAH were significantly lower in the DPSC-CM-treated group (Fig. 4B). In contrast, treatment with DPSC-CM significantly increased the mRNA expression levels of classical markers M2-like microglia secreting anti-inflammatory cytokines, TGF-β and Arg-1 (Fig. 4C, *P*  < 0.05 vs. SAH + Veh, respectively). Anti-inflammatory IL-10 protein levels in plasma were also significantly elevated in the SAH + DPSC-CM group (Fig. 4D, *P* < 0.001 vs. SAH + Veh). Furthermore, the downregulation effects on IL-1β and TNF-α and upregulation effects on Arg-1 and IL-10 were significantly reversed by the neutralizing anti-IGF-1 antibody.

### DPSC-CM Reduces Swelling of Astrocytes and AQP4 Expression in Primary Co-cultures of Astrocytes and Microglia Exposed to Hemolysate

**Fig. 5:**
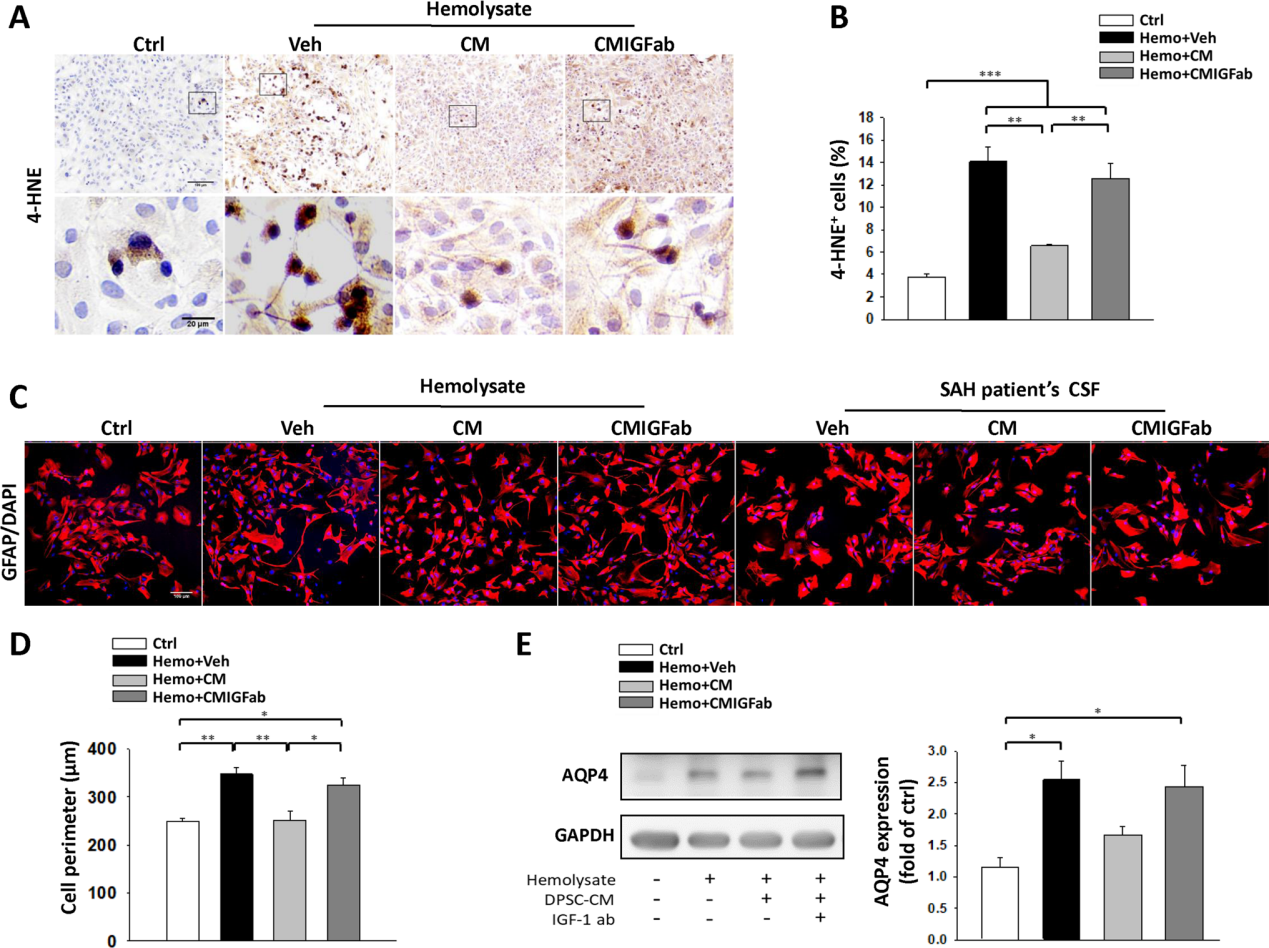
Effects of DPSC-CM on primary astrocytes exposed to hemolysate and SAH-patient CSF for 24 h. **A** Immunocytochemical staining for 4-HNE (shown in brown; upper panels, bar = 100 μm; lower panels, bar = 20 μm). **B** There was a significant decrease in the number of 4-HNE-positive cells in the hemolysate + DPSC-CM group. **C** Immunofluorescence staining for GFAP (shown in red). Representative images showing the effects of exposure of astrocytes to hemolysate or SAH-patient CSF with Veh/DPSC-CM/DPSC-CMIGFab treatment for 24 h, respectively (bar = 100 μm). **D** Quantitative analysis of GFAP^+^ cells showed that the astrocyte cell perimeter significantly increased after exposure to hemolysate for 24 h. DPSC-CM treatment significantly decreased the astrocyte cell perimeter. **E** The level of AQP4 protein in co-cultured astrocytes and microglia was measured by western blot. Hemolysate caused an increase in AQP4 expression, which was suppressed by DPSC-CM treatment. Data are expressed as means ± SEM. *P < 0.05, **P < 0.01, ***P < 0.001, *n* = 3

We next examined the effect of DPSC-CM on astrocyte swelling in primary co-cultures of astrocytes and microglia exposed to hemolysate (1 mg/ml) or SAH-patient CSF (10%) after 24 h of incubation. To monitor the oxidative stress level, immunocytochemical analysis of 4-HNE was performed (Fig. 5A). After quantification, hemolysate treatment led to a substantial increase in 4-HNE-positive cells as compared to control (*P* < 0.001 vs. Ctrl), while DPSC-CM treatment effectively ameliorated hemolysate-induced 4-HNE-positive cells in astrocyte/microglia co-cultures (Fig. 5B, *P* < 0.01 vs. hemolysate). To monitor astrocyte swelling, the astrocytic volume and morphology were evaluated by immunofluorescence staining for GFAP (Fig. 5C). A markedly swollen morphology of GFAP^+^ cells was observed in the hemolysate and SAH-patient CSF-treated group but not in DPSC-CM treated group. Astrocyte volume changes as indicated by GFAP^+^ cell perimeter measurements showed that hemolysate treatment significantly induced astrocyte swelling (Fig. 5D, *P* < 0.01 vs. Ctrl). In contrast, treatment with DPSC-CM decreased cell volume induced by hemolysate, (Fig. 5D, *P* < 0.01 vs. hemolysate). However, the effects were reversed by the neutralizing anti-IGF-1 antibody. Western blotting analysis showed both hemolysate stimulation and treatment in the DPSC-CM + anti-IGF-1 protein groups significantly increased the AQP4 protein expression level as compared with the control group (Fig. 5E, *P* < 0.05). However, the expression of AQP4 did not differ between the DPSC-CM treatment and control groups (Fig. 5E).

### DPSC-CM Promotes Microglial M2 Polarization Through AKT Activation in Primary Co-cultures of Astrocytes and Microglia Exposed to Hemolysate

**Fig. 6:**
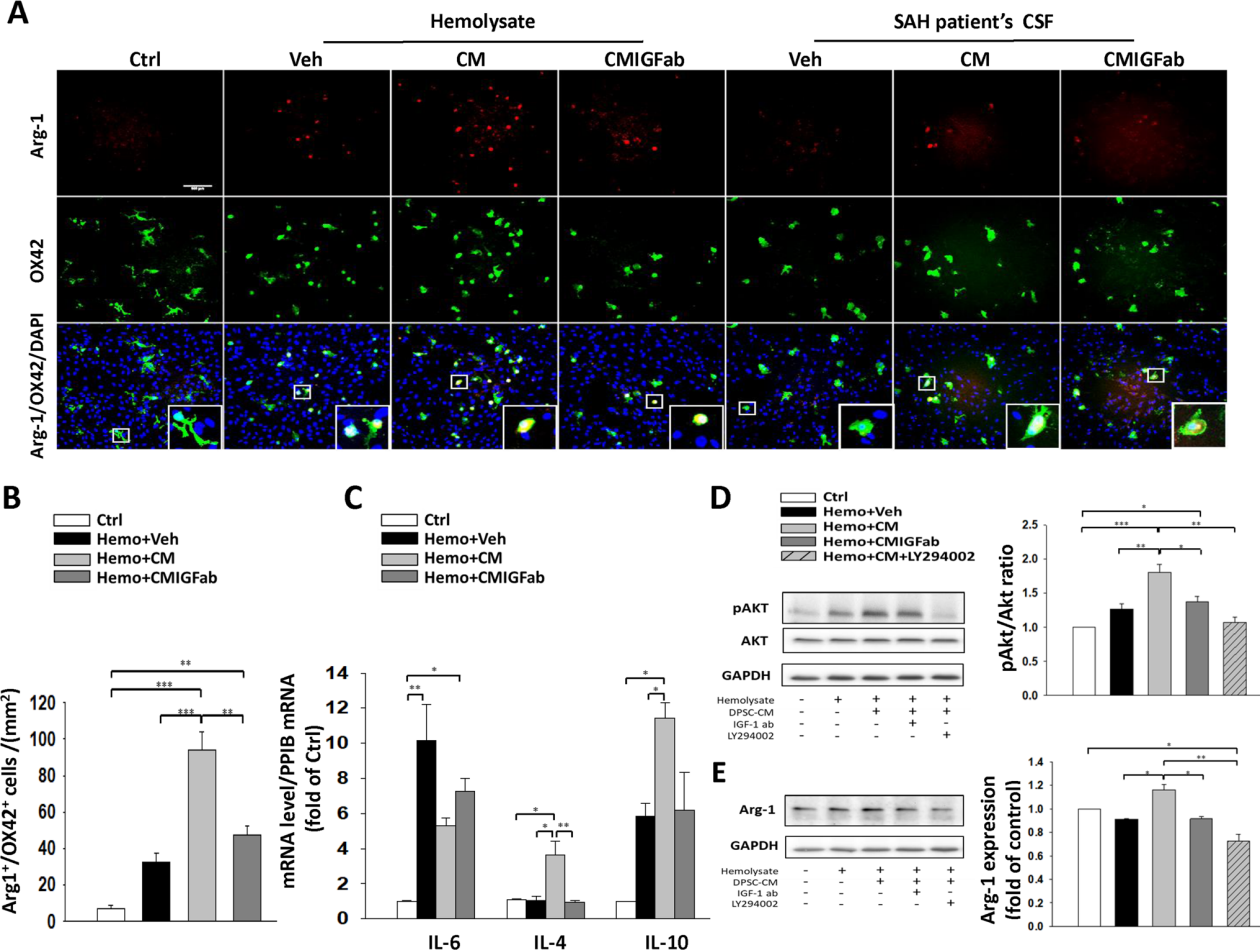
Effects of DPSC-CM treatment on microglial M2 polarization in astrocyte/microglia co-cultures exposed to hemolysate or SAH-patient CSF for 24 h. **A** Representative immunofluorescence images of Arg-1 and OX42 labeling in the astrocyte/microglia co-cultures. OX42 (a marker for activated microglia) immunoreactivity is shown in green, and Arg-1 (M2a microglia marker) is shown in red (bar = 100 μm). **B** Neutralization of IGF-1 moderately blunted the DPSC-CM-mediated microglia M2 polarization and decreased the number of Arg-1/OX42-positive cells after exposure to hemolysate. **C** Real-time PCR analysis showed that DPSC-CM markedly inhibited IL-6 and increased the IL-4 and IL-10 mRNA levels after exposure to hemolysate. Western blots and quantification showed that DPSC-CM treatment increased **D** pAKT (serine-473)/AKT ratio and **E** the expression of Arg-1 after exposure to hemolysate, which were both reversed by the neutralizing IGF-1 antibody and Ly294002 (AKT-PI3K inhibitor). Data are expressed as means ± SEM. *P < 0.05, **P < 0.01, ***P < 0.001, *n* = 2–3

To further confirm the therapeutic effects of DPSC-CM on microglia-mediated neuroinflammation after SAH, we evaluated the expression of M2 cell marker Arg-1 in activated microglia induced by hemolysate or SAH-patient CSF (Fig. 6A). The results of double immunofluorescence staining demonstrated that the DPSC-CM treatment group had a relatively higher level of Arg-1^+^/OX42^+^ cells than the Veh-treated group at 24 h following exposure to hemolysate or SAH-patient CSF (Fig. 6A). Neutralization of IGF-1 moderately blunted the DPSC-CM-mediated microglia M2 polarization and decreased the number of Arg-1^+^/OX42^+^ cells as compared with the DPSC-CM treatment group (Fig. 6B, *P* < 0.01). Moreover, treatment with DPSC-CM significantly decreased the mRNA expression level of pro-inflammatory cytokine IL-6 and increased the classical markers of M2-like microglia secreting anti-inflammatory cytokines IL-4 and IL-10 as compared to hemolysate + Veh group (Fig. 6C). In addition, Akt has been shown to participate in growth factor-induced macrophage M2 polarization [37, 38]. To investigate the role of IGF-1 in Akt activation, primary co-cultures of astrocytes and microglia were incubated hemolysate with DPSC-CM, DPCS-CM + IGF1ab, or LY294992 (an Akt-PI3K inhibitor). Western blotting analysis showed that treatment with DPSC-CM resulted in an increase in pAkt/AKT ratio (*P* < 0.01) and Arg-1 (*P* < 0.05) expression as compared with the Veh-treated group after exposure to hemolysate (Fig. 6D, E). However, the effects were reversed by exposure to IGF-1 neutralizing antibody and LY294002. Taken together, these findings indicated that IGF-1 with DPSC-CM plays an important role in regulating microglial M2 polarization following hemolysate-induced neuroinflammation through IGF-1/Akt signaling.

## Discussion

In the present study, we demonstrated that DPSC-CM treatment has therapeutic effects on brain edema and microcirculation impairment in the early phase of experimental SAH. DPSC-CM alleviated the obstructed capillaries, which were dramatically compressed by swelling of astrocyte end-feet in the cortex, and greatly improved reduced cortical blood flow and brain oxygen pressure at 24 h after SAH induction. Considering the important effects of the microglia-mediated inflammatory response and oxidative stress on astrocyte swelling, the present study demonstrated potential therapeutic effects of DPSC-CM treatment, with emphasis on targeting immunomodulatory functions and antioxidant properties.

Astrocytes swell rapidly in early brain injury after acute SAH, and this cytotoxic edema has emerged as a strong predictor of a poor functional outcome [39]. Previous studies showed that the neuroinflammatory response may initiate failure of water channel homeostasis, leading to intracellular edema [40]. Evidence shows that AQP4 deficiency reduces cytotoxic edema, astrocyte swelling, and pro-inflammatory cytokine secretion [41]. Another study indicated that post-ischemic cytotoxic brain edema was reduced in AQP4-knockout mice as compared with wild-type mice [42]. Yang et al. reported that overexpression of AQP4 in brain glial cells accelerated cytotoxic brain swelling by employing a transgenic approach in mice [43]. Our results showed that increased number of astrocytes (GFAP-positive cells) is not only accompanied by hypertrophy of the astrocytes but also increased expression of AQP4 and pro-inflammatory cytokines. Therefore, it is important to confirm that neuroinflammation is mainly attributed to microglia and astrocytes, which are cells specific to the CNS. Besides, a clinical study has shown that an immediate increase in the plasma level of the product of lipid peroxidation, 4-HNE, was observed after 24 h in SAH patients, especially in the case of severe brain edema, hematoma, and vasospasm complications [44]. Astrocyte swelling is correlated with oxidative stress, which contributes largely to brain edema [45], these abnormalities are in line with increased 4-HNE immunoreactivity in astrocytes, as shown in the present study. Furthermore, we found that the increase in brain water content paralleled 4HNE, GFAP, AQP4 protein levels, and several pro-inflammatory cytokine IL-6, IL-1β, and TNF-α mRNA expressions in the cortex region at 24 h following SAH, which were significantly alleviated by DPSC-CM treatment and these beneficial effects were abrogated by IGF-1 neutralizing antibody. Similarly, AQP4 expression were also observed to increase in response to the hemolystate-induced pro-inflammation and oxidative stress in the primary astrocyte/microglia co-cultures. Nevertheless, our finding showed that DPSC-CM treatment increased anti-inflammation cytokine IL-4, IL-10 expression and decreased 4-HNE-pocitive cells, which might contribute to decreased levels of AQP-4 expression. However, the protect effect was also blunted by IGF-1 neutralizing antibody in the in vitro model of SAH. The finding suggested that DPSC-CM substantially contributed to the treatment effect in terms of improving brain edema and microcirculation might through IGF-1 signaling mediated anti-inflammatory and antioxidant mechanism in SAH rats.

Microglia respond to brain injury through polarization of the M1 phenotype, which secrete pro-inflammatory proteins that have been found to be strongly associated with cerebral edema in the early phase of SAH [46]. Experimental evidence indicates that the pro-inflammatory factors IL-6, IL-1β, and TNF-α were significantly expressed by M1 microglia activation in an animal model of SAH [47]. It has been found that immunomodification of MSCs to express IL-4 could promote M2 microglia/macrophage polarization, functional recovery, and inhibit neuroinflammatory responses after brain injury [48, 49]. In addition, a deficiency of IL-4 accompanied a lower expression of IL-10, which is an M2 microglia marker and a potent anti-inflammatory cytokine produced by microglia, in a cerebral ischemia–reperfusion animal model [50]. Accumulating evidence indicates that IL-10 has anti-inflammatory and neuroprotective effects, and is also a key mediator of the crosstalk between neurons, astrocytes, and microglia [51, 52]. Consistent with previous findings, our results showed that DPSC-CM treatment contributed to elevation of anti-inflammation markers (IL-4 and IL-10) and suppression of pro-inflammation markers (IL-6, IL-1β, and TNF-α) in in vivo and in vitro models of SAH. Furthermore, immunofluorescence staining of Arg-1^+^/OX42^+^ and GFAP^+^ revealed that DPSC-CM promoted M2 microglial transformation and inhibited the increase in astrocyte volume in both hemolysate-treated and SAH-patient CSF-treated primary astrocyte/microglia co-cultures; however, the beneficial effects were not observed in the IGF-1 neutralizing antibody treatment group.

Systemic recombinant IGF-1 administration has been shown to significantly attenuated ischemia injury-induced microglial activation, cerebral edema, and improved sensorimotor function [53, 54]. IGF-1 treatment significantly influences IL-10 production in human T cells and rat plasma [55, 56], which was consistent with our finding that treatment with IGF-1 neutralizing antibody blunted the effect of DPSC-CM on the expression of IL-10 in rat plasma after SAH induction (Fig. 4D). In addition, IGF-1 is involved in signaling protein AKT phosphorylation, which is an important factor controlling microglia polarization of M1/M2 phenotypes and the expressions of antioxidant enzymes in the brain [37, 38]. One previous study demonstrated that a deficit in IGF-1/Akt signaling is related to a reduced ability to induce bone marrow macrophage M2 polarization, and the effect of IL-4 on Arg-1 protein expression was blunted by exposure to IGF-1 neutralizing antibody [57]. Correspondingly, following treatment with neutralizing antibody IGF-1, our in vitro data indicated that the levels of IL-4, IL-10, and M2 specific marker Arg-1 in microglia were reduced according to pAKT/AKT signal reduction, while the LY294002 (AKT-PI3K inhibitor) reversed the DPSC-CM effects on expression of Arg-1 following hemolysate treatment. This might explain the possible mechanism of the anti-inflammation effect of DPSC-CM via IGF-1/AKT pathway in terms of ameliorating astrocyte swelling and microcirculation impairment.

There are several limitations remain in this study. First, DPSC-CM was administered at 10 min before the SAH induction since intrathecal injections of autologous blood for SAH induction might increase intracranial pressure (ICP). Accordingly, the optimal therapeutic time window is needed to further validate. Second, transgenic construct in cell culture or animals could help elucidate causal relations between IGF-1 signaling and AQP4. Meanwhile, the other mechanisms of DPSC-CM underlying the IGF-1 signaling mediated anti-oxidative stress require further investigation. Third, besides IGF-1, DPSC-CM contains the TIMP-1 and TIMP-2 which were associated with inhibition of matrix metalloproteinase (MMP) protein levels, thereby restoring BBB integrity and improving cerebral edema following ischemic stroke [58, 59]. We cannot exclude the contribution of TIMPs in the DPSC-CM involved in attenuating microcirculation impairment after SAH. Nevertheless, DPSC-CM might be a better therapeutic agent as compare to single recombinant IGF-1 treatment but further work is required to investigation in SAH models.

## Conclusion

**Fig. 7:**
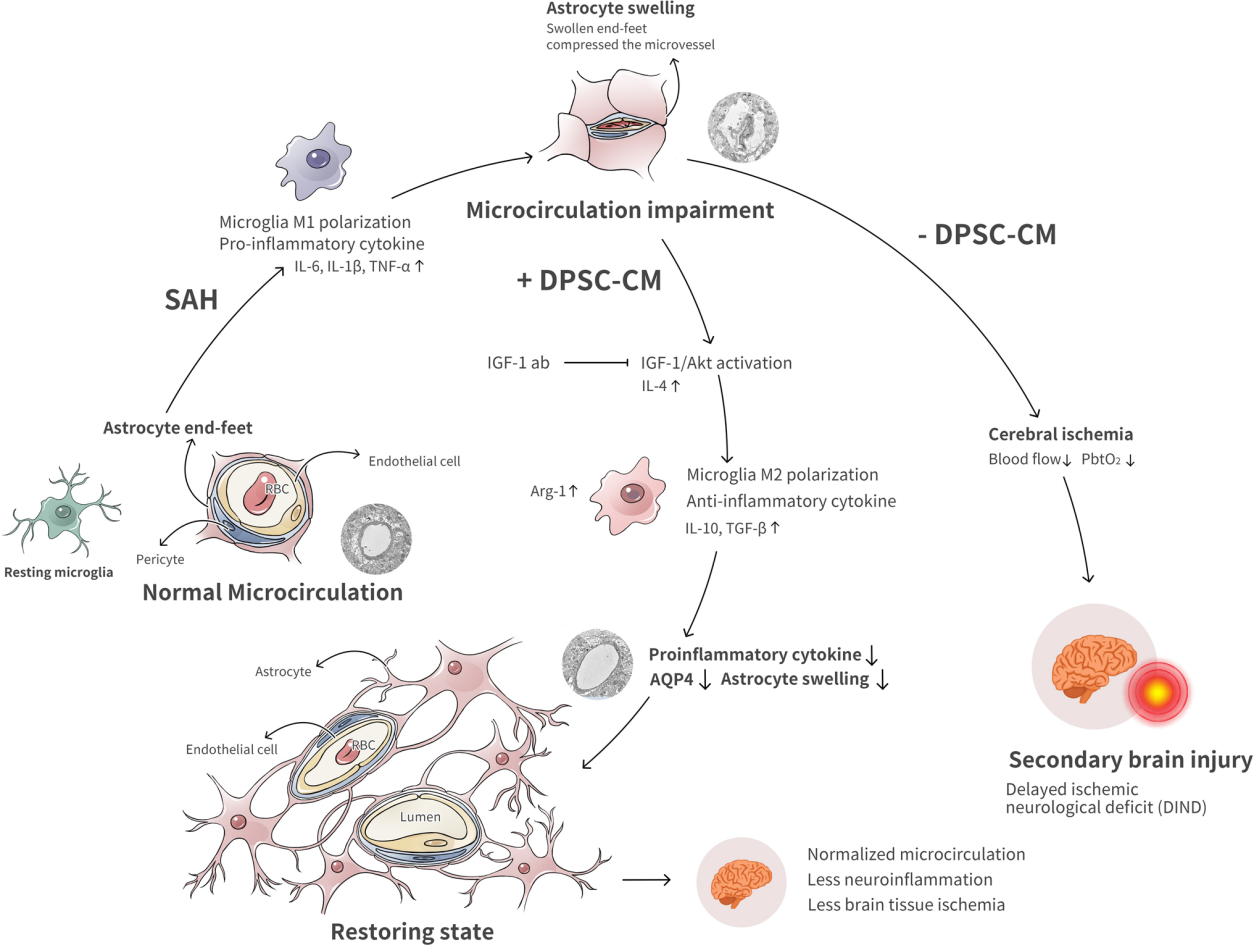
DPSC-CM restored SAH-induced astrocyte swelling-mediated microcirculation impairment and promoted microglia M2 polarization via the IGF-1/AKT pathway

Taken together, the main findings of the present study provide evidence regarding the possible mechanisms of the DPSC-CM therapeutic effect in promoting M2 microglia polarization to restore brain edema-mediated microcirculation impairment after SAH by partly depending on IGF-1/Akt signaling (Fig. 7). Examination of the therapeutic effects of DPSC-CM in the proposed future study will reveal novel insights into the control of early brain edema formation and immunomodification in the early phase after SAH, and might exploit the maximal beneficial effect of DPSC-CM and facilitate the development of a novel therapeutic strategy for other neurological disorders.

## Supplementary Information

Below is the link to the electronic supplementary material.

Supplementary file1 (DOCX 13 KB)

**Supplementary Figure 1. Effects of high-dose and low-dose DPSC-CM on microcirculation at 24 h after SAH. **(A) The regional cerebral blood flow and (B) the partial pressure of oxygen (PbtO_2_) at the brain surface were both significantly higher in the SAH+CM (40ul) rats than in the SAH+Veh rats. However, the blood flow and PbtO_2_ in the SAH+CM (20ul) rats did not show difference with the SAH+Veh rats. Data are expressed as means ± SEM. *P < 0.05, **P < 0.01, ***P < 0.001, n = 4-5.

High Resolution Image (TIF 199 KB)
