# Supplementary material for: Dental Pulp Stem Cell-Derived Conditioned Medium Alleviates Subarachnoid Hemorrhage-Induced Microcirculation Impairment by Promoting M2 Microglia Polarization and Reducing Astrocyte Swelling
Source: Transl Stroke Res. 2022 Oct 1;14(5):688–703. doi: 10.1007/s12975-022-01083-8 (PMC10444696; doi:10.1007/s12975-022-01083-8)
Supplement: Supplementary file 1 — Supplementary file1 (DOCX 13 KB) [file 12975_2022_1083_MOESM1_ESM.docx]

| **Supplementary Table 1. List of qRT-PCR primers and sequences.** | | |
| --- | --- | --- |
| **Target Gene** | **Forward Primer (5′-3′)** | **Reverse Primer (5′-3′)** |
| IL-6 | TGGAGTTCCGTTTCTACCTGGA | GAGCATTGGAAGTTGGGGTAGG |
| IL-1β | GCACTGCAGGCTTCGAGATG | AGGCCACAGGGATTTTGTCG |
| TNF-a | CGAGTGACAAGCCCGTAGCC | AGATAAGGTACAGCCCATCTGC |
| Arg1 | AGATGTGGACCCTGGGGAAC | CCCGTAGCCGGGGTGAATAC |
| TGF-β | ACCGGAGAGCCCTGGATACC | GTTGGCATGGTAGCCCTTGG |
| IL-4 | GCAACAAGGAACACCACGGAGAAC | CTTCAAGCACGGAGGTACATCACG |
| IL-10 | GAAGGACCAGCTGGACAACA | CTGCCTGGGGCATCACTTCT |
| Ppib | GCACGTGGTTTTCGGCAAAG | TTGGCAATGGCAAAGGGTTT |
