# Supplementary figures and images for: Dental Pulp Stem Cell-Derived Conditioned Medium Alleviates Subarachnoid Hemorrhage-Induced Microcirculation Impairment by Promoting M2 Microglia Polarization and Reducing Astrocyte Swelling
Source: Transl Stroke Res. 2022 Oct 1;14(5):688–703. doi: 10.1007/s12975-022-01083-8 (PMC10444696; doi:10.1007/s12975-022-01083-8)

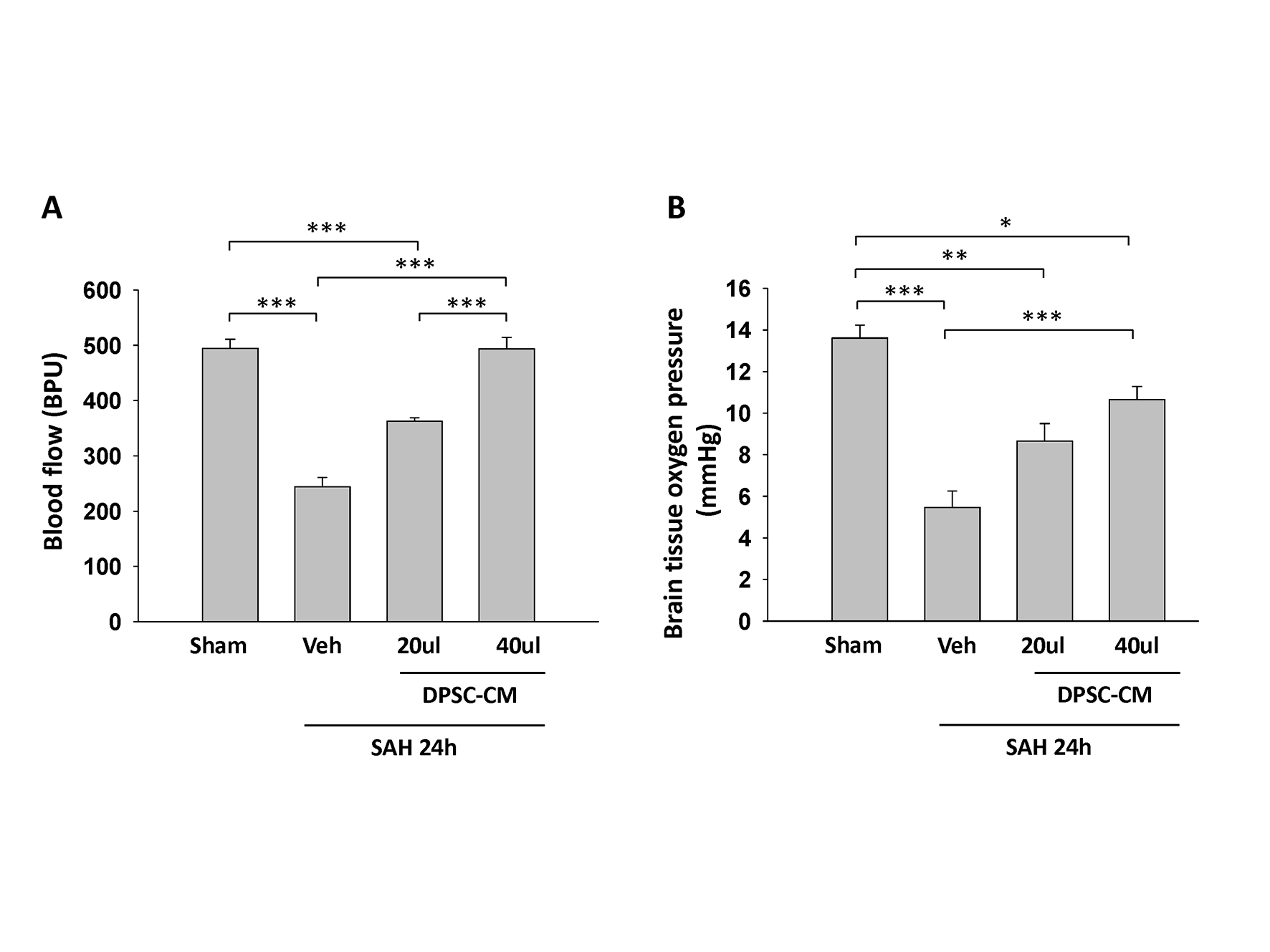

Supplement: Supplementary file 2 — Supplementary Figure 1. Effects of high-dose and low-dose DPSC-CM on microcirculation at 24 h after SAH. (A) The regional cerebral blood flow and (B) the partial pressure of oxygen (PbtO2) at the brain surface were both significantly higher in the SAH+CM (40ul) rats than in the SAH+Veh rats. However, the blood flow and PbtO2 in the SAH+CM (20ul) rats did not show difference with the SAH+Veh rats. Data are expressed as means ± SEM. *P < 0.05, **P < 0.01, ***P < 0.001, n = 4-5. [file 12975_2022_1083_Fig8_ESM.png]

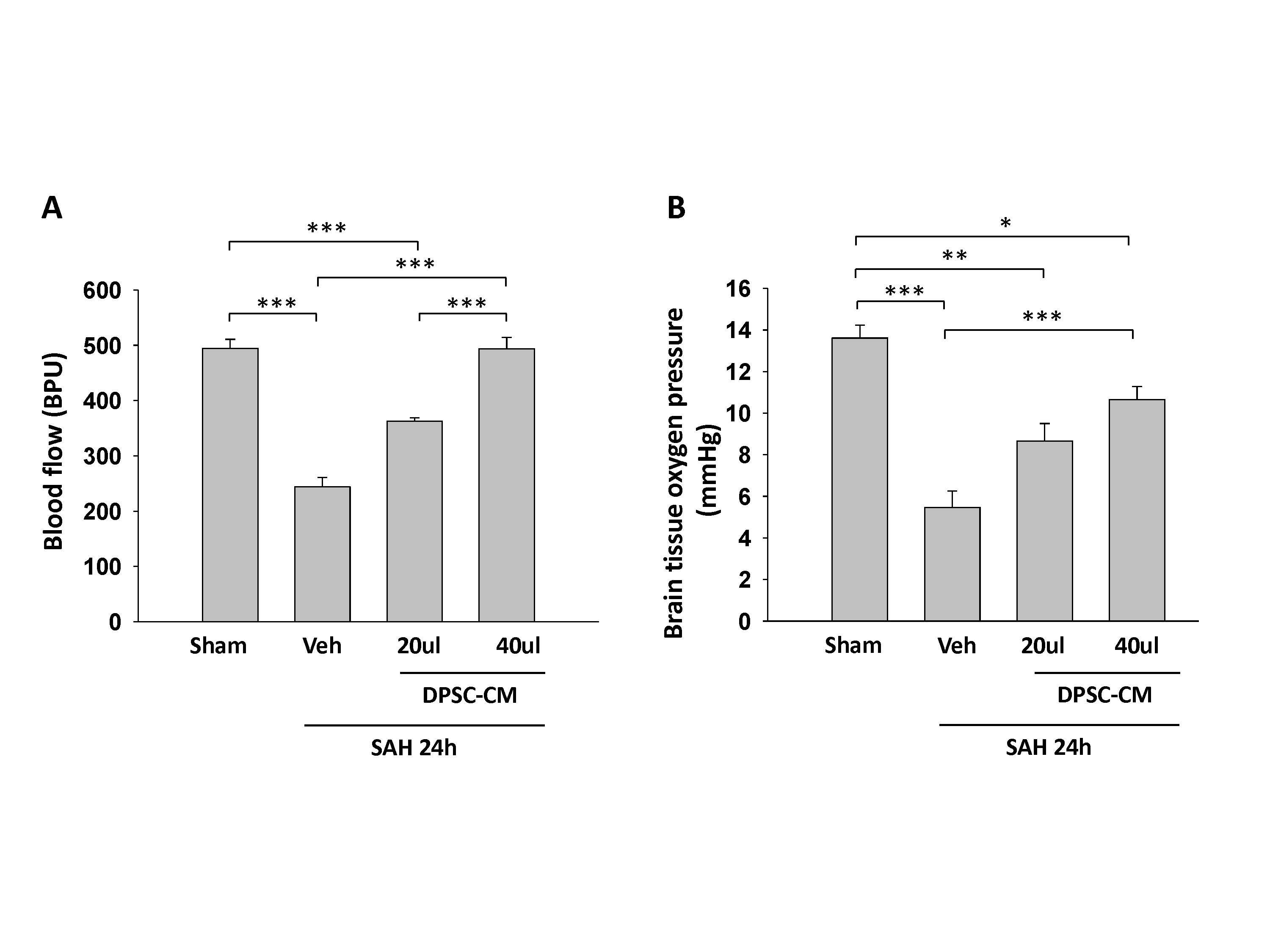

Supplement: Supplementary file 3 — High Resolution Image (TIF 199 KB) [file 12975_2022_1083_MOESM2_ESM.tif]
